# Network Reconstruction and Modelling Made Reproducible with moped

**DOI:** 10.3390/metabo12040275

**Published:** 2022-03-22

**Authors:** Nima P. Saadat, Marvin van Aalst, Oliver Ebenhöh

**Affiliations:** 1 Institute of Quantitative and Theoretical Biology, Heinrich-Heine-Universität Düsseldorf, Universitätsstraße 1, 40225 Düsseldorf, Germany; nima.saadat@hhu.de (N.P.S.); marvin.van.aalst@hhu.de (M.v.A.); 2 Cluster of Excellence on Plant Sciences (CEPLAS), Heinrich-Heine-Universität Düsseldorf, Universitätsstraße 1, 40225 Düsseldorf, Germany

**Keywords:** metabolic networks, modeling, topological networks, metabolic network expansion, network reconstruction

## Abstract

Mathematical modeling of metabolic networks is a powerful approach to investigate the underlying principles of metabolism and growth. Such approaches include, among others, differential-equation-based modeling of metabolic systems, constraint-based modeling and metabolic network expansion of metabolic networks. Most of these methods are well established and are implemented in numerous software packages, but these are scattered between different programming languages, packages and syntaxes. This complicates establishing straight forward pipelines integrating model construction and simulation. We present a Python package moped that serves as an integrative hub for reproducible construction, modification, curation and analysis of metabolic models. moped supports draft reconstruction of models directly from genome/proteome sequences and pathway/genome databases utilizing GPR annotations, providing a completely reproducible model construction and curation process within executable Python scripts. Alternatively, existing models published in SBML format can be easily imported. Models are represented as Python objects, for which a wide spectrum of easy-to-use modification and analysis methods exist. The model structure can be manually altered by adding, removing or modifying reactions, and gap-filling reactions can be found and inspected. This greatly supports the development of draft models, as well as the curation and testing of models. Moreover, moped provides several analysis methods, in particular including the calculation of biosynthetic capacities using metabolic network expansion. The integration with other Python-based tools is facilitated through various model export options. For example, a model can be directly converted into a CobraPy object for constraint-based analyses. moped is a fully documented and expandable Python package. We demonstrate the capability to serve as a hub for integrating reproducible model construction and curation, database import, metabolic network expansion and export for constraint-based analyses.

## 1. Introduction

Theoretical analysis of metabolic pathways has a longstanding tradition. The early approaches to study glycolysis, for example, have considerably increased our understanding of fundamental regulatory principles in metabolism [1]. In recent approaches, metabolic modeling was employed to study metabolic interdependencies in microbial communities and to identify putative drug targets for microbial pathogens [2,3].

Several theoretical techniques to study metabolism have been established. The most classic technique is the analysis of metabolic networks by representing them as systems of ordinary differential equations (ODEs). This representation heavily depends on detailed knowledge of stoichiometries, parameters of enzyme kinetics and regulatory mechanisms of reactions [4]. This approach is extremely useful for investigating relatively small systems. The upsurge of novel high-throughput experimental “omics” techniques led to the collection of immense amounts of data, resulting in an ever-increasing number of fully sequenced genomes. The improved quality of annotated genes resulted in a tremendous increase in information of enzymes and the respective metabolic reactions. This information has been collected in biochemical databases such as MetaCyc, BioCyc, KEGG or BiGG [5,6,7,8]. Such databases provide information for large-scale metabolic networks of many different organisms. However, analyzing such large-scale metabolic networks using systems of ordinary differential equations is difficult. This is, to a large extent, due to missing information on kinetic parameters of the involved enzymatic reactions [9]. One convenient alternative is constraint-based modeling and its mathematical method flux balance analysis (FBA) [10]. This commonly used approach uses the stoichiometric matrix of a reaction network and finds a steady-state vector of reaction fluxes that maximizes or minimizes an objective function that linearly depends on the reaction rates. Other structural analysis techniques focus on the topology of metabolic networks [11]. One such technique is metabolic network expansion and the related concept of metabolic scopes. The metabolic scope describes the set of metabolites, which are topologically producible by a given network from an initial set of compounds [12,13]. Thus, metabolic network expansion allows to functionally characterize metabolic networks with respect to their biosynthetic capacities [14].

Topological techniques are extremely useful in the process of curating models, in particular to identify and add missing reactions [15]. This process, called gap filling, allows, for example, to complement draft metabolic networks in order to guarantee that observed compounds can be produced from the growth medium [16].

Many of the techniques described above have been implemented as Python packages. However, most of these software packages are not directly compatible with each other.

In this work, we present moped, a compact but useful Python package that serves as a hub, offering tools for analysis, development and extension or modification of metabolic models. The integration of BLAST and pathway/genome databases such as MetaCyc and BioCyc into moped allows reconstructing metabolic network models directly from genome sequences [17] and ensures that the reconstruction process is fully transparent and reproducible. In addition to the de novo construction of models, moped provides an interface to import existing metabolic network models in SBML format.

To facilitate curation of metabolic models, moped provides an interface to Meneco, a topological gap-filling tool based on answer set programming [18]. All models created with moped can easily be exported as CobraPy objects, thus directly integrating constraint-based with model construction and modification [19]. It is even possible to extract a scaffold model of metabolic pathways for kinetic modeling via modelbase [20]. The Python package moped presented here is a mathematical modeling hub, which allows constructing reproducible metabolic models de novo, integrating existing models in SMBL format, curating models by gap filling and performing topological or constraint-based analyses.

## 2. Implementation

### 2.1. Model Import, Extension and Modification

moped uses SBML files or PGDB flat files as input for constructing a metabolic network model. PGDBs are organism-specific pathway/genome databases containing annotated reactions and compounds of the metabolism of the organism [6]. These databases further include detailed information about reactions and compounds, such as sum formulas, charges, references to other database entries or subcellular localization. This information is of great importance for a consistent analysis of metabolic networks. SBML files represent metabolic networks in an XML-based format and can be considered as a standard for the exchange of reconstructed and curated metabolic models between tools and platforms [21]. Such files can be, among others, obtained from databases such as BiGG, which provides SBML files of curated metabolic models together with information about the corresponding publications [7].

**Figure 1:**
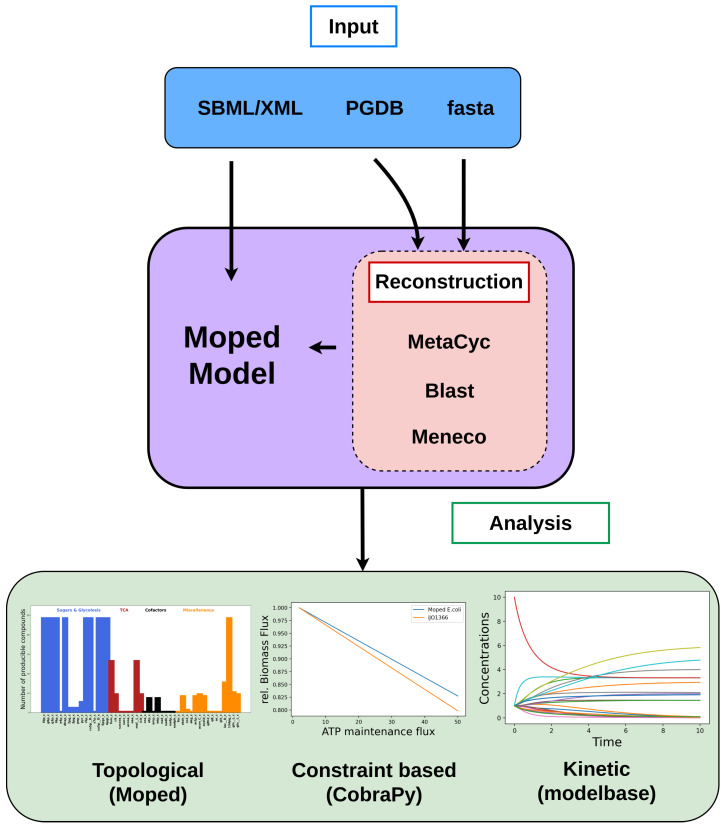
The modeling hub moped. moped accepts SBML, FASTA files or MetaCyc and BioCyc PGDBs as inputs. PGDBs and SBML files are directly converted into a moped object. By BLASTing genome/proteome-sequences against MetaCyc, moped models can be constructed utilizing GPR rules. Further reconstruction can be achieved using Meneco for gap filling. Topological model analysis is implemented in moped. For constraint-based and kinetic analysis, moped offers export as CobraPy and modelbase objects, respectively [19,20].

Because of the wide range of import methods (FASTA, PGDBs and SBML), one particular strength of moped is the integration of several analysis tools. An overview of mopeds functionalities is shown in Figure 1. Furthermore, moped provides an accessible environment to extend or modify constructed or imported models. Therefore, adding alternative or additional metabolic pathways to pre-existing models, as well as modifying compound and reaction identifiers, is simple and straightforward. Naturally, all moped objects can be exported as SBML. A UML diagram of moped can be found in Figure S1 in the Supplementary Material.

### 2.2. Tools for Metabolic Network Expansion

**Figure 2:**
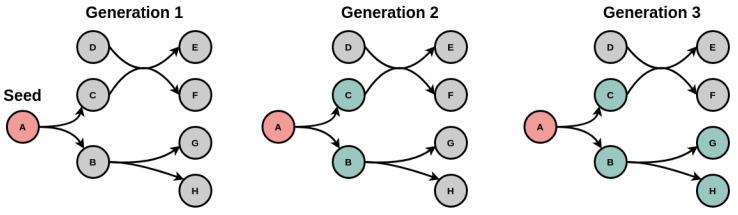
Metabolic network expansion. Beginning with an initial set of compounds, the seed (here panel 1), the expansion process detects all producible compounds in a network and adds them to the seed for the next generation until no additional producible compounds are found.

A useful and valuable feature of moped is the fully implemented network expansion algorithm to perform metabolic network expansions on moped objects. Metabolic network expansion can be used to investigate structural properties of metabolic networks, such as biosynthetic capacities and their robustness against structural perturbations [12]. The core concept of metabolic network expansion is the metabolic scope, which contains all compounds that are producible by a network from a given initial set of compounds, termed the seed (see Figure 2). In the expansion process, the seed is used to find all reactions that can proceed in their annotated direction. The respective products are then added to the seed, forming the new seed for the next expansion step. This process continues until no new compounds can be added to the seed. Thus, scopes characterize biosynthetic capacities of metabolic networks, based exclusively on their topology.

**Figure 3:**
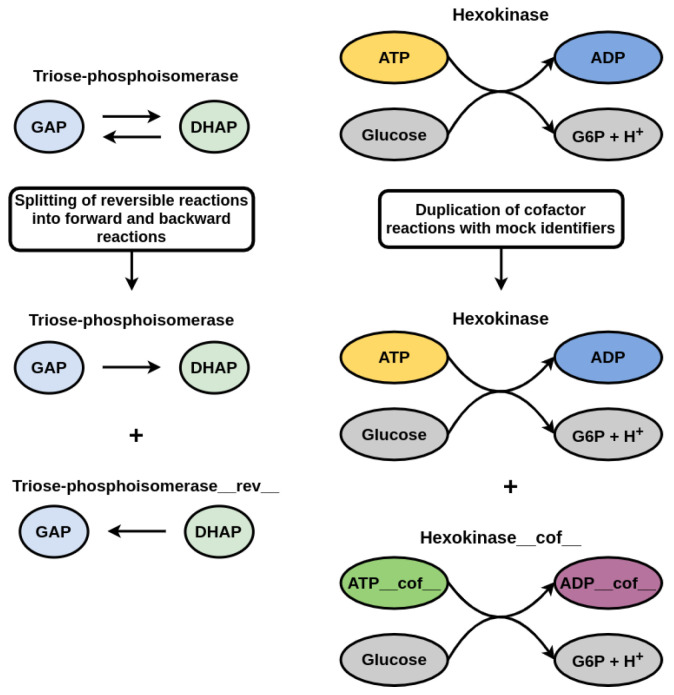
Topological network modifications moped offers functions for splitting reversible reactions into forward and backward reactions in a network. Adding a copy of each cofactor dependent reaction and replacing cofactors (here ATP and ADP) with mock identifiers allows unblocking cofactor dependent reactions while avoiding degradation products of cofactors contained in the seed. Such modifications enable biologically feasible metabolic network expansion.

Network expansion depends on a precise definition of reaction reversibilities and involved cofactors. Network expansion uses the stoichiometry of reactions to identify producible compounds. However, stoichiometric coefficients of reactions are annotated for one particular direction. To include the opposite direction (for reversible reactions) into the metabolic network expansion, moped provides a method for reversibility duplication. As illustrated in Figure 3 for triose-phosphoisomerase as an example, this method finds all reversible reactions in a moped object and adds the reversed reaction to the network. The new reaction identifier is identical to the identifier of the original reaction concatenated with the suffix ‘ rev ’. This model modification can be reverted if no longer needed.

Many reactions depend on specific cofactors. Cofactors usually appear in pairs. One of the most prominent examples is the cofactor pair ATP and ADP. In the majority of reactions with ATP as substrate, ATP serves as a donor of a phosphate group, thus producing ADP. Only a few reactions actually modify the adenosine moiety (for example, in nucleotide de novo synthesis). In network expansion, therefore, no reaction utilizing ATP or ADP as cofactor could proceed, unless these compounds are either included in the seed or can be produced from metabolites within the seed. If the purpose of network expansion is to realistically calculate a set of producible compounds, this behavior is not desired because it leads to a drastic underestimation of the scope. The most naive approach to directly include cofactors in the seed yields misleading results, because in such a case, all compounds that can be generated from digesting, e.g., ATP would be included in the scope.

A pragmatic approach to solve this problem is the duplication of cofactors as proposed in [12]. Here, reactions with cofactor pairs are duplicated, where the copied reactions contain “mock cofactors”. In contrast to the real cofactors, the mock cofactors only occur in reactions, in which they act in their role as cofactors. For ATP, this is the transfer of a phosphate group, for NADH or NADPH the transfer of protons and electrons and for acetyl coenzyme-A, the transfer of the acetyl group. The cofactor duplication allows the use of mock cofactors inside the initial seed. Reactions depending on cofactors might now be able to occur in the expansion process. However, reactions using the cofactors as proper substrates can only occur if the real cofactor can be produced from the seed.

moped provides a convenient method for finding and duplicating all reactions using cofactor pairs. The cofactor pairs can either be automatically determined by moped for networks imported from BiGG or MetaCyc (see Tables S1 and S2 in the Supplementary Material) or they can be declared individually by the user. The identifiers of the duplicated cofactors are replaced by mock identifiers, which contain the suffix ‘ cof ’. The same modification is applied to the respective reaction identifiers. This model modification can be reverted if no longer needed.

The implemented methods for cofactor and reversibility duplication are commonly used to obtain biologically meaningful results for metabolic network expansion. However, they are also highly useful for topological gap-filling using Meneco, during model reconstruction. This is further explained in the next section.

### 2.3. Reconstruction of Draft Network Models

Construction of metabolic networks highly depends on reliable databases. In order to enable user-friendly metabolic network reconstruction, moped includes methods for importing data from the MetaCyc and BioCyc databases, identifying homologous sets of genes with BLAST and gap-filling.

MetaCyc is a universal, highly curated reference database of metabolic pathways and biochemical reactions from all domains of life. BioCyc is a database of organism-specific PGDBs containing metabolic network information based on predictions by the PathoLogic component of the Pathway Tools software [22,23]. The MetaCyc and BioCyc databases provide many advantages. Both databases are freely available for academic and nonprofit users. All PGDBs are available in useful flat file formats. Furthermore, these databases include information on the reaction directions based on experimental references and thermodynamics, extensive annotations and, therefore, information about gene–protein–reaction (GPR) associations, as well as thermodynamic information about metabolites and reactions such as the Gibbs energy of formation and the standard Gibbs energy of reactions.

In order to use BioCyc and MetaCyc for metabolic network construction and analysis, moped offers a parser for PGDBs, allowing direct construction of moped objects from MetaCyc or BioCyc flat files. moped objects can directly be used for network analyses including network expansion and constraint-based modeling. Especially for the latter, it is extremely important that all reactions are mass- and charge-balanced to ensure that all solutions obey mass conversation. Therefore, only reactions which are mass- and charge-balanced are parsed in moped. While this process has the danger of omitting annotated genes, including reactions that are not mass- or charge-balanced would violate fundamental physical principles and lead to unrealistic model properties. This pipeline provided by the database import and parsing of moped makes it straightforward to construct prokaryotic network models. For eukaryotic metabolic networks, however, intensive and careful curation is required due to missing compartment information. More detailed information about the parsing of PGDBs using moped can be found in the documentation.

There exist several pipelines to automatically extract a set of metabolic reactions from a genome or proteome sequence. One popular pipeline is the above mentioned PathoLogic software. moped integrates such a pipeline into the Python programming language, directly converting a genome/proteome sequence into a moped object that can be immediately used for modeling applications. This functionality is provided by an implemented wrapper for local BLAST to find enzyme reactions in genome sequence fasta files or proteome fasta files by similarity search against enzyme reference sequences from the MetaCyc database. This method constructs a new moped object representing a metabolic network of all reactions that are found in a genome sequence or proteome using enzyme monomer amino acid sequences and protein–reaction annotations from MetaCyc to ensure fulfilled gene–protein–reaction associations (GPRs) in all found reactions [24]. This process can be controlled by user-defined thresholds. This integrated pipeline makes the model reconstruction perfectly reproducible and illustrates the functionality of moped as a modeling hub.

The next curation step after the initial automatic network construction is usually gap-filling. This describes a process in which reactions are added to the network in order to ensure that the reconstructed model reflects experimentally observed behavior, such as the production of experimentally measured compounds from the growth medium [25]. There are many available gap-filling methods such as GapFill or MIRAGE [26,27]. Most of these methods are based on constraint-based approaches. A common problem is that these approaches can predict gap-filling solutions that use thermodynamically infeasible cycles. In this sense, these approaches are sensitive to self-producing or energy-generating cycles [18]. Meneco, in contrast, is a topological gap-filling tool based on the network expansion algorithm. Meneco calculates a minimal set of reactions that need to be added to a draft network such that a specified list of target compounds can be produced from a given set of seed compounds. This gap-filling approach offers the advantage that it is inherently impossible for gap-filling solutions to depend on infeasible cycles. Meneco gap-filling can be directly applied as a method to moped objects. One moped object represents the draft network and a second the repair network, from which the added reactions are chosen.

The topological network modifications, i.e., reversibility and cofactor duplication, harmonize ideally with the application of Meneco, resulting in networks with biologically realistic behavior. This again illustrates the integrative nature of the modelling hub moped. For an accurate manual curation, automatically determined gap-filling reactions should always be manually inspected before adding them to the network model.

A major advantage and distinguished feature of moped is the complete reproducibility of the construction of draft models, which is much needed in systems biology, and the subsequent manual curation [28]. In moped, the user can add and remove reactions, or even entire pathways, from draft networks. Furthermore, the user can inspect the reactions found by Meneco to fill gaps and decide if these reactions are valid for specific models. All user decisions become part of an executable Python script, making them perfectly reproducible by others. This underlines that in moped, early curation can be integrated closely into the draft model reconstruction process. The importance of such reproducibility and traceability has recently been highlighted [29]. To our knowledge, this feature is unique to is unique to moped and is not yet found in any other reconstruction software. For constraint-based modeling, the user can define which exchange reactions are to be included and, if desired, define their own specific objective functions. moped offers a template biomass function which is based on the iJO1366 and iML1515 biomass functions (see Table S3 in the Supplementary Material); however, users should be encouraged to design their own specific and precise biomass function for their models as a part of correct manual curation. Reconstructing draft networks in moped lays the ground for model curation without the need to change software environments. In all reconstruction and curation steps, user decisions are documented as commands in an executable Python script, thus making them fully reproducible and transparent.

## 3. Results

### 3.1. Displaying the Advantage of Cofactor Duplications in Topological Network Analysis

**Figure 4:**
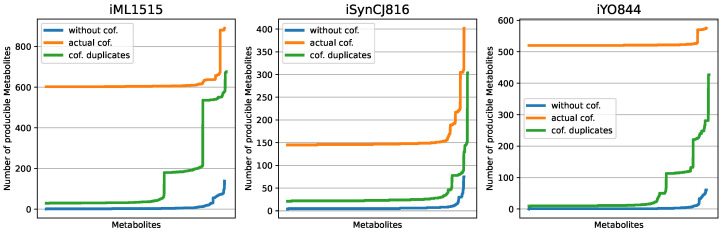
Metabolic scopes in established models of *E. coli* (iML1515), *B. subtilis* (iYO844) and *Synechocystis* sp. PCC 6803 (iSynCJ816). The differently colored graphs represent the same analysis but including no cofactors, actual cofactors and cofactor duplicates in the seed.

To display the benefits of including the moped cofactor duplication, three established models of *E. coli*, *B. subtilis* and *Synechocystis* sp. PCC 6803 have been parsed into moped for a comparative metabolic network expansion [30,31,32]. In this analysis, we calculated all single metabolite scopes (i.e., the scopes for the seed consisting only of a single metabolite and water) for the respective models. This has been done in three variations: (i) including no cofactors to the seed, (ii) including the original cofactor compounds and (iii) including on the mock cofactors resulting from cofactor duplication (see above). Figure 4 displays the scope sizes (number of compounds contained in the scope) for each model and each variant to calculate the scopes. Apparently, without cofactors, the scopes are small for most compounds (blue lines). This can be explained by the missing connectivity for reactions that require cofactors. The analysis including the actual cofactor compounds in the seed (orange lines) displays an unrealistically large metabolic scope for every compound, even for inorganic metabolites. This can be explained by the fact that cofactors are usually rather complex metabolites, and now all degradation processes are included during the network expansion. Therefore, the resulting metabolic scopes are no longer reflecting the property of the compound of interest but rather the degradation products of the metabolized cofactor compounds. The corresponding analysis of models using cofactor duplication and mock cofactors duplicates in the seed (green lines) demonstrates that for small or inorganic metabolites, the scope is still relatively small. For more complex organic compounds, the metabolic scope is increasing without artificially increasing the scope size with degradation products of cofactors. This demonstrates the perks of including cofactor duplication and mock cofactors in seeds for biologically more realistic metabolic network expansions.

### 3.2. Applying Metabolic Network Expansion to a Model of *E. coli* Core Metabolism

We illustrate moped’s metabolic network expansion algorithm with a compact network of *E. coli* core metabolism, which is freely available in SBML format from the BiGG database [33]. After importing the SBML file into moped, we applied cofactor and reversibility duplications as described above.

**Figure 5:**
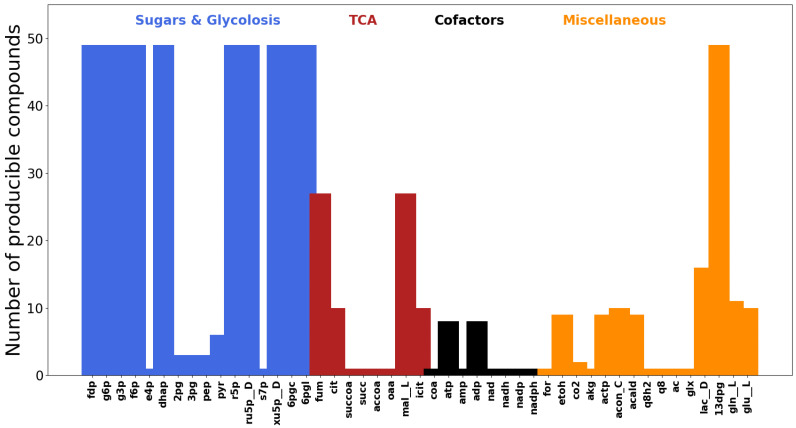
Metabolic scopes of all compounds in the *E. coli* core metabolic model calculated using moped. The *Y*-axis indicates the total amount of compounds producible from every compound, water and a set of acceptor mock cofactors.

For each metabolite in the network, we calculate the scope size, i.e., how many new compounds are producible if only this metabolite, water and a set of mock cofactors are available. The results of that analysis are displayed in Figure 5. In this relatively small metabolic network (72 metabolites and 95 reactions), eleven key compounds, which are mostly part of central metabolism, exhibit a largest observed scope size of 47. Such detailed metabolic network expansion is useful to provide insight about central metabolites, as well as structural and functional characteristics of metabolic networks [14]. Whereas we here only display the scope size, the methods implemented in moped allow a far wider spectrum of analysis methods, including determination the set of producible metabolites, as well as following each step of the expansion process. The code used to produce the results and Figure 5 can be found on https://gitlab.com/marvin.vanaalst/moped-publication-2021/-/tags/final-publication, accessed on 13 December 2021.

### 3.3. Comparison of Draft Reconstructions with Established Models and Softwares

We demonstrate how moped provides a complete and easy-to-use pipeline to construct genome scale models from genome and proteome sequences and how these models can be directly applied for constraint-based analyses. For this, we download the freely available proteome sequences of *Escherichia coli* str. K-12 substr. MG1655, *Synechocystis* sp. PCC 6803 and *Bacillus subtilis* strain 168 [34,35,36]. We import the MetaCyc PGDB to construct a moped object of the MetaCyc database as a reference network. Applying the BLAST wrapper, which was described above, to the FASTA files and the reference network, we obtained three moped objects, representing the draft network reconstructions. Then, we applied gap filling to ensure that the reconstructed models can produce all basic biomass compounds (inspired by the *E. coli* biomass reaction from iJO1366 [37], including all nucleic acids, amino acids and lipid precursors) from M9 minimal glucose medium. For this analysis, we directly accepted all resulting gap-filling reactions. For a more accurate reconstruction, the proposed gap-filling reactions should be manually inspected before addition to the draft model. We added exchange reactions for all medium compounds and tested if the draft models can exhibit a stationary flux distribution to produce biomass, as determined by flux balance analysis. The construction of these models can be reproduced using the notebooks provided on our accompanying git.

**Figure 6:**
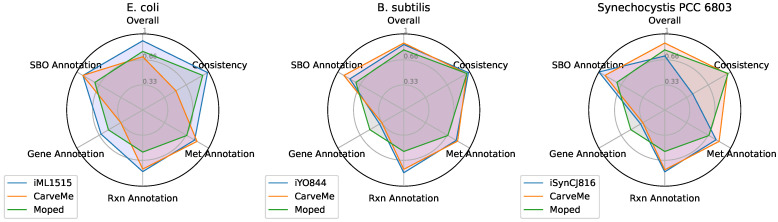
MEMOTE evaluations for draft model reconstructions produced by CarveMe and moped, as well as established models, for *E. coli*, *Bacillus subtilis* and *Synechocystis* sp. PCC 6803. MEMOTE evaluations include the stoichiometric consistency and the annotation level of models.

In order to test the quality of our draft models, we compared them with established models for the respective organisms (iML1515, iYO844 and iSynCJ816) [34,35,36]. Furthermore, we used the same dataset and medium to construct draft models with the established genome scale modeling reconstruction software CarveMe [38]. In order to quantitatively compare all three versions of the organism network reconstructions, we used metabolic model testing (MEMOTE) pipeline to establish a fair and reproducible comparison [39]. MEMOTE calculates scores for genome scale metabolic models to evaluate the stoichiometric consistency, the GPR rules and the quality of annotations for reactions and metabolites in the respective models. A summary of the MEMOTE evaluations for the three models for the three organisms is presented in Figure 6. The MEMOTE evaluation shows that the stoichiometric consistency of draft models produced by moped is always of high quality. Figure 6 shows that draft models reconstructed by CarveMe and moped display generally good overall scores and annotations. While CarveMe draft model reconstructions show the tendency to provide better reaction annotations, moped draft model reconstructions display a generally better annotation of genes and GPR rules.

**Figure 7:**
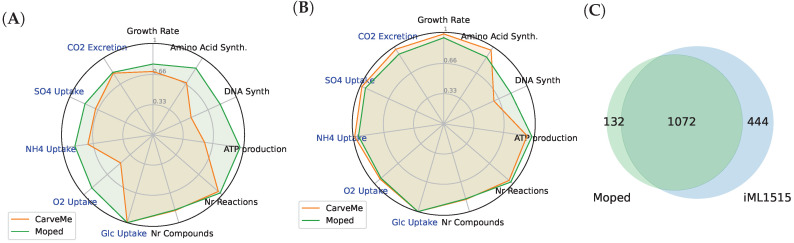
Functional comparison of the draft model reconstructions using moped and CarveMe with iML1515. We calculated maximal growth rates, respective ATP production rates and exchange fluxes for compounds in the medium, as well as optimal production rates for amino acids and nucleic acids for completely unmodified draft models (**A**) and models with identical biomass functions and reaction bounds (**B**). In the radar plots, the relative distance between the two values are reported. Panel (**C**) shows the overlap of GPR annotations found in the draft model constructed with moped and iML1515.

The functionality and predictive power of draft models constructed by moped has been compared for *Escherichia coli* str. K-12 substr. MG1655 with a similarly constructed draft model using CarveMe, and the iML1515 model. For this analysis, the models automatically constructed moped and CarveMe were analysed without further modification. We calculated maximal growth rates, respective ATP production rates and exchange fluxes for compounds in the medium. Furthermore, we calculated optimal production rates for amino acids and nucleic acids. These model functionalities have been compared to the predictions of iML1515. Figure 7A displays the predicted fluxes of the draft models constructed by moped and by CarveMe relative to the predictions of iML1515. In the radar plots, the relative distance is indicated. For two flux values $v_{1}$ and $v_{2}$, the distance $\min(v_{1}/v_{2},v_{2}/v_{1})$ is plotted. The draft model constructed with moped shows a higher similarity to the behaviour of iML1515 in almost all functionalities, especially in oxygen uptake rate, ATP production rate and nucleic acid synthesis. Some discrepancies between the model behaviours can be linked to slightly differing biomass compositions and lower bounds for exchange fluxes. In order to reduce such bias, we performed the same analysis but with such adjustments that biomass compositions and all lower and upper bounds are identical. Extended MEMOTE evaluations can be found in Figure S2 in the Supplementary Material. Figure 7B shows that now draft models produced with moped and CarveMe exhibit very similar behaviour to iML1515 in all functionalities, except in nucleic acid synthesis, in which moped draft models are more similar to iML1515. The overlap of GPR annotations of the draft model constructed with moped and iML1515 is shown in Figure 7C. The vast majority of genes in the draft model constructed with moped can be found in iML1515 and therefore illustrates the quality of the automated reconstruction. This analysis has only been performed with the draft model constructed with moped because the draft model constructed with CarveMe and iML1515 do not share any common database links. These results shows that draft model reconstructions made with moped exhibit a high quality that is able to keep up with the quality of established models and software tools.

## 4. Conclusions

Here, we present moped, a Python package representing a hub connecting the construction, modification and curation of genome scale metabolic networks with various analysis methods, which support studies of metabolic networks. moped supports the de novo construction of metabolic networks by importing databases, providing homology searches, including GPR associations and integrating an established gap-filling routine without the need to change software environments. Existing models from external sources can be imported using the standardized format SBML. Metabolic network models are represented as moped objects, which can be modified by easy-to-use and intuitive methods. moped models can be exported into various formats, thus integrating a diverse set of established analysis tools. Metabolic network expansion and constraint-based optimization can be easily performed for any model represented as a moped object.

Examination of moped draft model reconstructions using MEMOTE demonstrated that the resulting models are generally of a high quality. The strength of draft model reconstructions with moped is the direct integration into the Python programming language: Every decision in the automatic and manual reconstruction process is documented in executable Python scripts. Therefore, the whole reconstruction process becomes fully transparent and is easily reproducible by any interested user.

The modular architecture of the open source package moped is particularly designed for allowing further extensions to enhance its functionality, such as the integration of additional software tools. We provide an extensive documentation for moped, as well as troubleshooting guides, unit-tests for all provided methods and example notebooks illustrating the usage of moped at https://gitlab.com/marvin.vanaalst/moped-publication-2021 (accessed on 13 December 2021).

## Data Availability

Operating systems: Linux, OS X Programming language: Python License: GPLv3 Any restrictions to use by nonacademics: For nonprofit use only All source code including scripts to produce all manuscript figures can be found at https://gitlab.com/marvin.vanaalst/moped (accessed on 13 December 2021) and https://gitlab.com/marvin.vanaalst/moped-publication-2021 (accessed on 13 December 2021).

## Supplementary Materials

The following are available online at https://www.mdpi.com/article/10.3390/metabo12040275/s1, Figure S1: UML diagram of core packages in moped, Figure S2: Extended MEMOTE evaluations for draft model reconstructions, Table S1: Cofactor pairs of MetaCyc identifiers, Table S2: Cofactor pairs of BiGG identifiers, Table S3: Default biomass composition

## Abbreviations

The following abbreviations are used in this manuscript:

PGDB	Pathway/Genome Database
FBA	Flux Balance Analysis
GPR	Gene–Protein–Reaction
SBML	Systems Biology Markup Language
ODE	Ordinary Differential Equations
