# Supplementary material for: Network Reconstruction and Modelling Made Reproducible with moped
Source: Metabolites. 2022 Mar 22;12(4):275. doi: 10.3390/metabo12040275 (PMC9032245; doi:10.3390/metabo12040275)
Supplement: Supplementary file 1 [file metabolites-12-00275-s001.zip › metabolites-1532142-supplementary.pdf]

Supplementary Material for Network reconstruction and modelling made reproducible with `moped`

Nima P. Saadat[1], Marvin van Aalst[1], and Oliver Ebenhöh[1,2,+]

[1] Institute of Theoretical and Quantitative Biology, University of Düsseldorf and CEPLAS-Cluster of Excellence on Plant Sciences, Düsseldorf, Germany

[2]Cluster of Excellence on Plant Sciences (CEPLAS), Heinrich-Heine-Universität Düsseldorf, Universitätsstraße 1, 40225 Düsseldorf, DE

[+]Correspondence to: oliver.ebenhoeh@uni-duesseldorf.de

1 Tables of Cofactor pairs

Table S1: Cofactor pairs of MetaCyc identifiers

**Strong Cofactor**	**Weak Cofactor**
ATP	ADP
GTP	GDP
NADH	NAD
NADPH	NADP
10-FORMYL-THF	THF
METHYLENE-THF	THF
5-METHYL-THF	THF
ACETYL-COA	CO-A
Donor-H2	Acceptor
Reduced-ferredoxins	Oxidized-ferredoxins
Red-NADPH-Hemoprotein-Reductases	Ox-NADPH-Hemoprotein-Reductases
Cytochromes-C-Reduced	Cytochromes-C-Oxidized
Plastocyanin-Reduced	Oxidized-Plastocyanins
ETF-Reduced	ETF-Oxidized

Red-Thioredoxin	Ox-Thioredoxin
CPD-12829	PLASTOQUINONE-9

Table S2: Cofactor pairs of BiGG identifiers

**Strong cofactor**	**Weak Cofactor**
atp	adp
gtp	gdp
nadh	nad
nadph	nadp
10fthf	thf
methf	thf
fdxrd	fdxox
trdrd	trdox
etfrd	etfox
accoa	coa
pcrd	pcox

2 Table of Biomass composition

The template biomass funtion in moped is a subset of all available compounds and respective stoichiometric coefficients from the established models iJO1366 and iML1515. We encourage users to design their own objective/biomass function in much needed manual curation steps.

Table S3: Default biomass composition

**Compound**	**Stoichiometric coefficient**
TRP_c	-0.055234
GLT_c	-0.255712
MALONYL-COA_c	-3.1e-05
GTP_c	-0.209121
NADP_c	-0.000112
WATER_c	-48.752916
LEU_c	-0.437778
ASN_c	-0.234232
L-ASPARTATE_c	-0.234232
L-ALPHA-ALANINE_c	-0.499149
ARG_c	-0.28742
TYR_c	-0.133993
THR_c	-0.246506
CTP_c	-0.129799
SER_c	-0.209684
ATP_c	-54.119975
GLN_c	-0.255712
MET_c	-0.149336
LYS_c	-0.333448

ACETYL-COA_c	-0.000279
CYS_c	-0.088988
HIS_c	-0.092056
VAL_c	-0.411184
UTP_c	-0.140101
ILE_c	-0.282306
NADPH_c	-0.000335
NAD_c	-0.001787
PRO_c	-0.214798
PHE_c	-0.180021
GLY_c	-0.595297
Pi_c	53.945874
PROTON_c	51.472
ADP_c	53.95

3 General Implementation and structure of Python object/classes

`moped` is a package fully integrated in the object-oriented programming language Python. The core is the `moped.Model` class, which instantiates a metabolic network from scratch or from input files like SBML or pathway/genome database (PGDB) flat files. This class includes reactions and compounds, which contain all extracted information for the respective attributes of the metabolic network. The `moped.analysis` module includes the module `moped.analysis.blast`, which constructs a `moped.Model` from all reactions in the MetaCyc database that can be found within genome/proteome sequences using BLAST and GPR rules. For this, `moped` requires FASTA files as input (genome or proteome), and parameters for BLAST can be specified. The module `moped.analysis.gapfilling` allows gap-filling via Meneco using one `moped.Model` object as the draft network and another one as the repair network. The last module, `model.analysis.scope` provides topological analysis of `moped.Model` objects using metabolic network expansion. The core classes are displayed in an unified modelling language graph in Figure 1. `moped` can be installed using `pip install moped==1.9.3`.

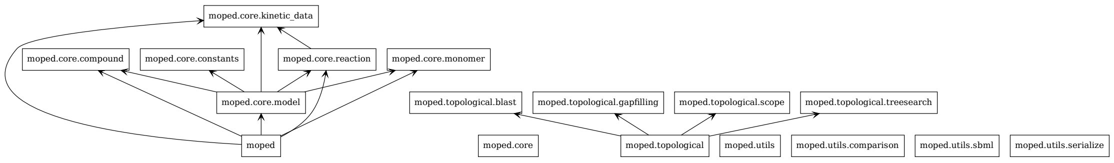

Figure S1: UML diagram of core packages in moped

4 Extended MEMOTE evaluations for draft model reconstructions, including Unbounded Flux analysis.

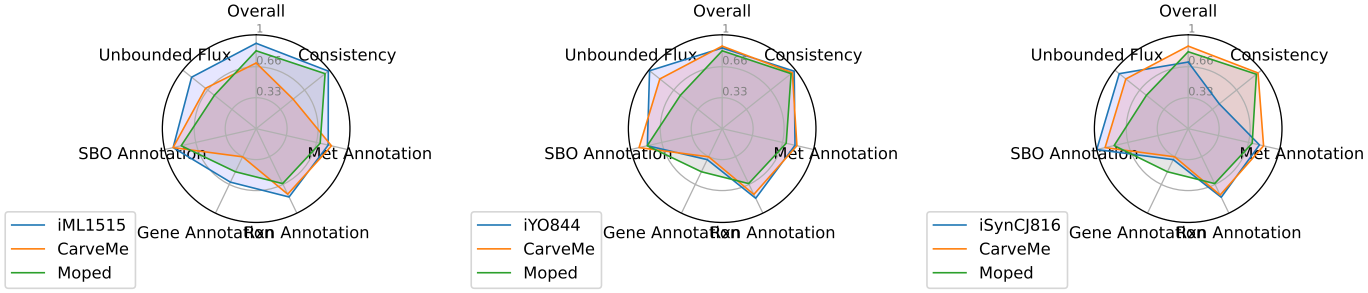

Figure S2: Extended MEMOTE evaluations for draft model reconstructions
